# Indirect Costs Arising From COVID-19-Related Work Absenteeism From October 2020 to July 2022: A Retrospective Study From Central Greece

**DOI:** 10.7759/cureus.81358

**Published:** 2025-03-28

**Authors:** Ioannis C Lampropoulos, Erasmia Rouka, Ioannis Pantazopoulos, Eleftherios Aggelopoulos, Dimitrios Papagiannis, Foteini Malli, Konstantinos I Gourgoulianis

**Affiliations:** 1 Department of Respiratory Medicine, University of Thessaly, Larissa, GRC; 2 Department of Nursing, University of Thessaly, Larissa, GRC; 3 Department of Business Administration, University of Patras, Patras, GRC

**Keywords:** central greece, cost of illness, covid-19, employers, indirect cost, pandemic, productivity loss, sick leave, social security

## Abstract

Background and objective

The coronavirus disease 2019 (COVID-19) pandemic has resulted in significant indirect costs due to the implementation of safety measures and work absenteeism in various sectors of the economy, thereby impacting productivity. This study aimed to examine the indirect costs of the recent pandemic in Thessalia, Central Greece, from October 2020 to July 2022.

Methods

This retrospective study focused on retail employees aged 20-70 years with absenteeism of 5-45 days. Indirect costs were assessed using administrative wage and leave data.

Results

During the study period, 1,400 employees were on sick leave: 971 women (69.36%) with a mean age of 44.4 ±9.67 years and 429 men (30.64%) with a mean age of 39.85 ±11.53 years. The study sample included 311 individuals with confirmed COVID-19. Workforce reduction averaged 2.65 workers per month, with estimated costs of €9,948.50 for employers and €90,214.58 for the National Social Security Institution.

Conclusions

These findings highlight substantial increases in labor costs incurred due to work absenteeism caused by the COVID-19 pandemic. Future research should explore clinical, demographic, and societal factors influencing COVID-19-related productivity losses, which would help inform strategic resource allocation policies.

## Introduction

The cost of illness (COI) is defined as "the value of the resources that are expended or forgone as a result of a health problem” [1]. Research on the economic burden of illness usually focuses only on direct healthcare costs, thus underestimating COI [1]. The coronavirus disease 2019 (COVID-19) pandemic has led to widespread work absenteeism on account of sick leaves, resulting in lost productivity and substantial indirect costs globally [2]. Investigations focusing on health indicators and COI in Greece and worldwide are critical to identify country-specific characteristics, thereby allowing for comparisons.

There have been 5,494,594 confirmed cases of COVID-19 reported in Greece, with 38,009 related deaths (latest update: December 19, 2023) [3]. Since the confirmation of the first severe acute respiratory syndrome coronavirus 2 (SARS-CoV-2) case on February 26, 2020 [4], three additional variants (alpha, delta, and omicron) of the virus have circulated in the country [5]. The “stay-at-home” guidance of the National Public Health Organization and quarantine requirements significantly affected the workplace situation. The exploration of workdays lost and indirect costs related to lost productivity due to safety and sick leave under current health protocols remains limited. Only one study has assessed both the direct and indirect costs associated with COVID-19 among healthcare personnel in Greece from the payer's perspective [6].

Given the significance of COVID-19's indirect costs on the health economy and the limited data on labor costs in Greece, this study aims to quantify the indirect costs of COVID-19-related work absences among retail workers in Central Greece, estimating employer and social security expenses while identifying key demographic and economic factors influencing productivity losses. By estimating productivity losses, the researchers seek to shed light on the concealed expenses linked to COVID-19, thereby enhancing the economic analysis of healthcare services. This understanding is pivotal for crafting strategic public health policies and making informed decisions regarding resource allocation.

## Materials and methods

Study design and population

A retrospective study was conducted from October 2020 through July 2022. This time period spanned the significant COVID-19 outbreaks in Greece. The research population included retail employees working in the private sector in Thessalia, Central Greece. The study was approved by the Research Ethics Committee of the University Hospital of Larissa (288/15.09.2022) and each participant provided written informed consent.

In Greece, the online sickness benefit service (e-EFKA) [7] covers workers' absences due to illness. This digital platform facilitates applications for these benefits, targeting individuals insured by e-EFKA who have accumulated at least 120 days of insurance coverage in the previous year or over the last 15 months, excluding the most recent three months. The study utilized data sourced from this registry. We defined the duration of leave as the period from the initial to the final day recorded for safety or sickness absence. The study focused on COVID-19-affected individuals aged 20-70 years, with absences ranging from 5 to 45 days. All COVID-19 cases were confirmed through the real-time reverse-transcription polymerase chain reaction (RT-PCR) method. Data validation was performed by cross-referencing e-EFKA records with employer-reported absences.

We calculated the indirect costs that businesses and the National Social Security Institution faced by looking at employee wages and the rates for safety or sick leave. During the study, the standard monthly pay was €650.00 for single full-time employees and €715.00 for those who were married. For part-time workers, who typically work 30 hours a week, we first adjusted their hours to match full-time work to determine their daily pay. Based on this, part-time wages were €23.40 per day for single employees and €25.74 for married ones. This breaks down to an hourly wage of €3.90 for single and €4.29 for married part-time workers. Under COVID-19 control and social protection laws, employees received half (50%) of their usual daily wage for the first three days of safety or sick leave. For the days that followed, they were paid the difference between their daily wage and the sickness benefit from the National Social Security Institution.

This sickness benefit is based on the insurance class of each employee, which is assigned based on the "imputed daily wage," and the benefit equals half of that. The "imputed daily wage" is calculated from the average earnings in the last 30 days of work in the year before the one the illness was reported (https://www.kepea.gr/) [8]. The imputed daily wage was set at €29.39 for married full-time workers and €26.76 for single ones. Part-time workers had an imputed daily wage of €26.76 if married and €24.11 if single. Finally, the average safety/sick pay rates were also calculated since the days of absence on official holidays are only paid by the National Social Security Institution and not by the employer.

Statistical analysis

The SPSS Statistics software v 26.0 (IBM Corp., Armonk, NY) was used to conduct the statistical analysis. Data distribution was assessed using the Kolmogorov-Smirnov normality test. Descriptive statistics are presented as mean ± standard deviation (SD). The Mann-Whitney U test and the Kruskal-Wallis test were used to determine significant differences in non-parametric data between two and more than two groups, respectively. Statistical significance was set at p<0.05. Figures were generated using Microsoft Excel 2016.

## Results

During the study period, 1400 employees were on sick leave: 971 women (69.36%) with a mean age of 44.4 ±9.67 years and 429 men (30.64%) with a mean age of 39.85 ±11.53 years. From October 2020 through July 2022, 311 workers out of 1,400 in the sample were reported as COVID-19 cases, amounting to an incidence rate of 22.2%, and these employees were included in our analysis. Twenty-three workers tested positive on two different occasions during the study (incidence rate: 7%; four males with a mean age of 47.75 ±8.58 years, and 19 females with a mean age of 45.58 ±8.70 years). A summary of cases with RT-PCR-confirmed SARS-CoV-2 infection per year and month is presented in Table 1.

**Table 1:** Reported cases with a positive RT-PCR test per year and month during the study period (October 2020-July 2022) RT-PCR: reverse-transcription polymerase chain reaction

	2020	2021	2022
January	-	2	5
February	-	6	5
March	-	12	5
April	-	17	4
May	-	11	6
June	-	4	3
July	-	6	62
August	-	8	-
September	-	14	-
October	15	37	-
November	41	34	-
December	6	8	-
Total	62	159	90

Of the 311 COVID-19 cases, 228 were women (73.31%) with a mean age of 45.49 ±8.10 years; of them, 184 were married, and 44 were single. The remaining 83 men (26.69%), of which one was married and 82 were unmarried, had a mean age of 39.47 ±9.94 years. Regarding the type of employment, 152 workers, of which 88 were married and 55 were single, had a part-time employment contract. The remaining 159 workers, of which 119 were married and 49 were single, had a full-time employment contract.

**Figure 1:**
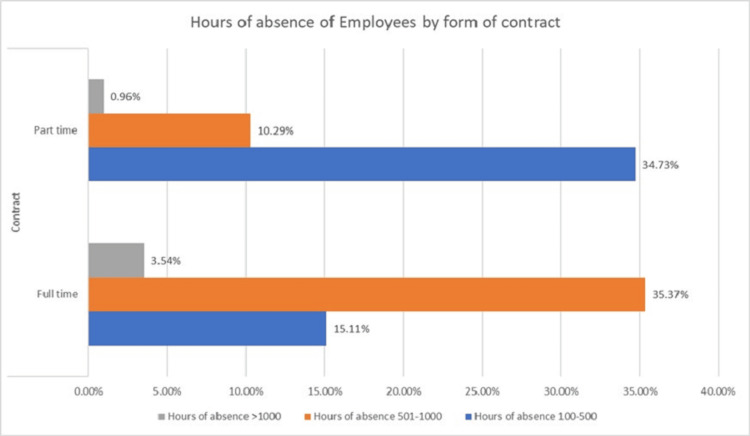
Hours of work loss by type of employment

When comparing the types of employment contracts in relation to the hours of work loss, a statistically significant difference was observed (U=5497.500; p<0.001) (Figure 1).

Table 2 presents the hours employees would have worked per year versus the actual working hours due to their absence. The minimum number of days of absence was five, and the maximum was 45 ±7.13, while the minimum number of hours of absence was 100, and the maximum was 1,560 ±279.73.

**Table 2:** Deviations in working hours for the period October 2020-July 2022 SARS-CoV-2: severe acute respiratory syndrome coronavirus 2

Year	Working hours as per contract	Actual working hours due to absence from SARS-CoV-2	Deviation in hours	Percentage deviation
2020	686,700	647,173	-39,527	-5,76%
2021	1,686,600	1,590,457	-96,143	-5,70%
2022	906,300	890,229	-16,071	-1,77%

The average (mean) number of days that workers were on leave was 17 for the years 2020 and 2021 and 5.33 for the year 2022. More specifically, the averages for the year 2020 were 17.12 ±5.46 days for women and 18 ±5.88 days for men. In the year 2021, for both men and women, the average was 17 days, while in 2022, it was 5.18 ±0.83 days and 5.38 ±1.51 days for men and women, respectively.

**Figure 2:**
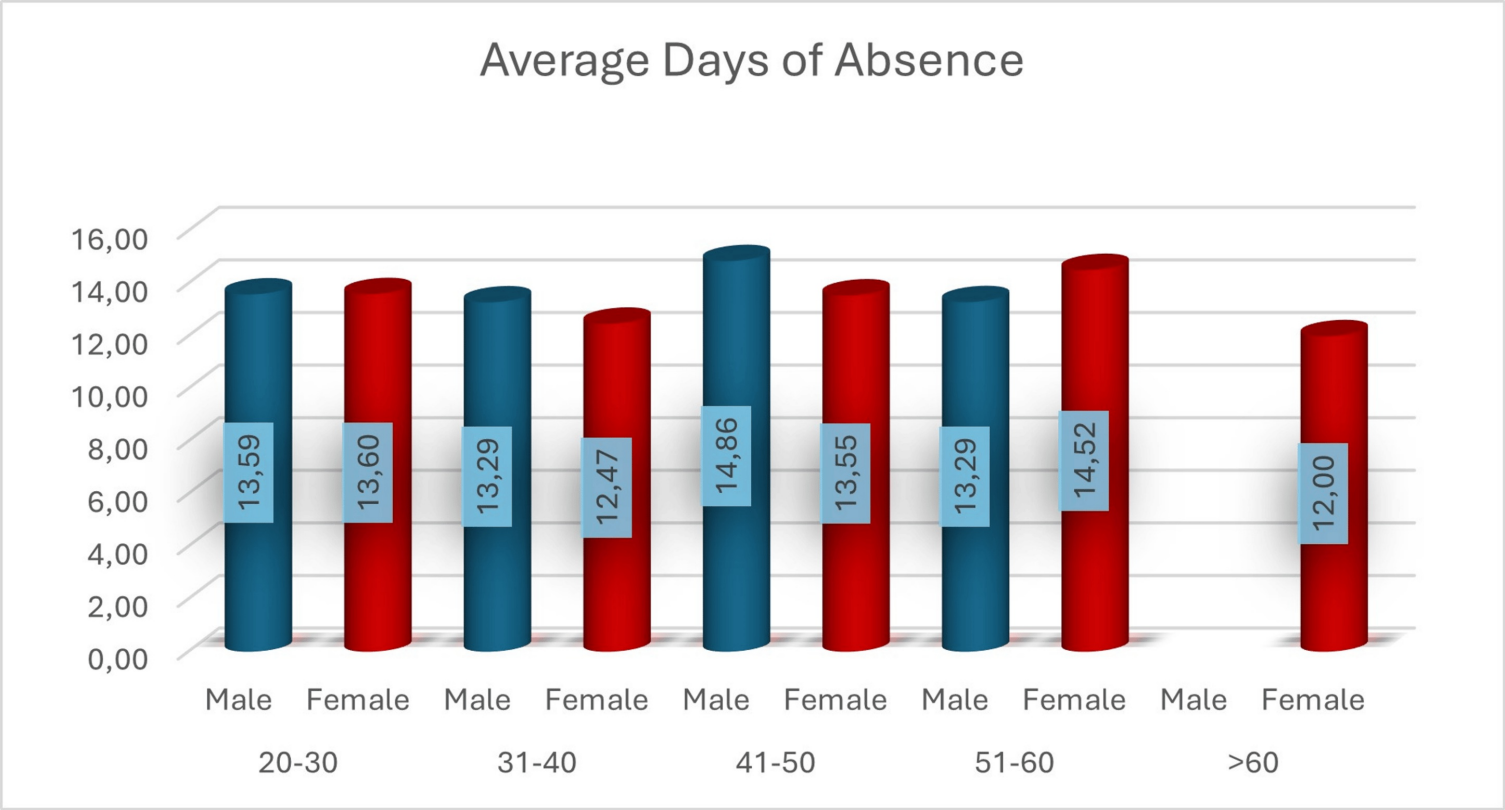
Workdays lost by gender and age category

Figure 2 shows the average (mean) number of workdays lost by gender and age category.

The details on days of work loss per month by type of employment are presented in Table 3.

**Table 3:** Days of work loss per month by type of employment

Year	Months	Days of absence - full-time employment	Days of absence - part-time employment
2020	October	197.00	54.00
	November	486.00	229.00
	December	73.00	27.00
2021	January	0.00	29.00
	February	76.00	50.00
	March	106.00	165.00
	April	108.00	263.00
	May	132.00	63.00
	June	29.00	29.00
	July	36.00	71.00
	August	82.00	45.00
	September	195.00	29.00
	October	341.00	229.00
	November	276.00	254.00
	December	22.00	67.00
2022	January	5.00	28.00
	February	5.00	20.00
	March	5.00	20.00
	April	0.00	20.00
	May	0.00	32.00
	June	10.00	5.00
	July	165.00	165.00
	Average	106.77	86.09
	Sum	2,349.00	1,894.00
	min	0.00	5.00
	max	486.00	263.00
	SD	124.84	84.53

The economic burden incurred during the 22-month study period was estimated at €9,948.50 for the employers and €90,214.58 for the National Social Security Institution (Table 4).

**Table 4:** Economic burden of COVID-19 for employers and insurance funds in Central Greece from October 2020 through July 2022 COVID-19: coronavirus disease 2019

Year	Months	Cost for employers, €	Cost for insurance funds, €
2020	October	462.37	6,008.73
	November	1,213.81	16,158.97
	December	187.52	2,286.11
2021	January	57.12	586.33
	February	170.08	2,987.17
	March	317.68	6,231.59
	April	477.41	8,698.56
	May	335.01	4,535
	June	117.95	1,201.81
	July	166.24	2,349.6
	August	247.88	2,837.57
	September	445.76	5,067.09
	October	1,138.95	12,446.71
	November	1,042.56	11,519.36
	December	248.8	1,697
2022	January	177.56	455.14
	February	183.76	267.6
	March	179.96	262.3
	April	143.39	208.78
	May	212.93	358.74
	June	107.73	155.26
	July	2,314.03	3,895.16

In the last three months of 2020, the cost for the organization amounted to €1,863.70, while the insurance funds were charged €24,453.81. In the year 2021, these costs amounted to €4,765.44 and €60,157.79, respectively, while in the first seven months of the year 2022, the cost for employers amounted to €3,319.36 and €5,602.98 for the insurance funds.

Based on these data, the average number of insurance days was three for each employee (311 employees x first three days = 933 days) plus 11.35 days for the remaining days. Therefore, for the 22-month study period, companies functioned with 2.65 employees less per month (3,7313.34 days/1,400 employees), which affected customer service.

## Discussion

The economic impact of COVID-19-related safety measures and work absenteeism on the adult working population and organizations in Greece has been largely overlooked until now. This study represents the first attempt to assess the indirect costs associated with the recent pandemic in the most populous geographical region of the country, which has 688,255 inhabitants according to the 2021 census [9].

The findings showed that the average workforce loss per month was 2.65 employees, with an estimated cost of €9,948.50 for the employers and €90,214.58 for the National Social Security system. The results also indicated a statistically significant difference in lost working hours between full-time and part-time employees. As shown in Figure 1, full-time employees experienced longer absences, which may be associated with their greater exposure time in the workplace. Although part-time employees also contracted the virus, their total hours of absence may have been lower due to their reduced weekly working hours. Our analysis further revealed that women accounted for 73.31% of the infected cases, with a mean age of 45.49 ±8.10 years, whereas men represented 26.69% of cases, with a mean age of 39.47 ±9.94 years. While the overall female representation in the sample was higher, their absence due to COVID-19 disproportionately increased.

As depicted in Figure 2, employees aged 41-50 years had the highest number of days of absence, which could be linked to increased occupational exposure or a greater likelihood of developing symptoms requiring prolonged recovery. Conversely, mean days of absence were lower for employees over 60 years old, as they either worked remotely or were granted special leave under protective measures. Our data suggest that COVID-19-related absences significantly impacted business operations, especially in the early months of the pandemic. Table 3 indicates that the months with the highest workday losses were November 2020 and October 2021, coinciding with major pandemic surges. Additionally, as seen in Table 4, the financial burden on employers and the social security fund was higher in 2021 - before the reduction in quarantine days in 2022.

By combining economic theory with infectious disease models, Xiang et al. [10] found that the COVID-19 pandemic had a direct negative impact on labor supply, output, and economic growth. Keyvanlo et al. [11] estimated that the total indirect cost of work absenteeism due to COVID-19 in northeastern Iran was $513,688 (approximately equal to €469.9). The authors suggested that the country's crisis management unit should design and implement preventive interventions for future epidemics. The consideration of preventive-oriented strategies was also proposed by Rajabi et al. [12] in their cross-sectional research conducted in Bushehr province, Iran. The researchers estimated that COVID-19-related indirect costs were $3,081.44 (approximately equal to €2,819) per patient.

More recently, Sell et al. [13] reviewed the available literature on the economic burden of COVID-19 for employers and employees in the United States. The authors concluded that most COVID-19 cases impact working-age adults and that productivity loss is higher among unvaccinated individuals. Ose et al. [14] surveyed nine northwestern European countries to assess the burden and responsibility sharing between the social protection system, employers, and workers. The authors reported different trends between countries, the majority of which adjusted their sick-pay system and increased sick leave coverage during the pandemic, except for the Netherlands and Belgium. Greece's indirect costs are possibly higher due to stricter sick leave policies. Thus, local policymakers should consider employer compensation mechanisms to mitigate future productivity losses.

Vardavas et al. [15] conducted a systemic review to assess the cost of the COVID-19 pandemic and the cost-effectiveness of mitigation interventions in countries of the European Union (EU), the European Economic Area (EEA), the UK, and the Organization for Economic Co-operation and Development (OECD). The researchers found that the economic burden of the COVID-19 pandemic is substantial and that cost-effective strategies to deal with the disease are dependent on population vaccination and the effective reproduction number at the stage of the pandemic.

A systematic literature review [16] on the economic burden of seasonal influenza among adults aged 18-64 years suggested that the estimation of indirect costs is associated with significant uncertainty, especially for absenteeism‐related data, as these are often collected from patient surveys or doctors' certificates and not from insurance databases or employers' records. The substantial productivity burden of influenza and influenza-like illness was recently highlighted by Blanchet Zumofen et al. [17]. An assessment of the methodological quality of economic evaluations of measles outbreaks pointed out the need for robust reporting guidelines [18].

The scarcity of scientific data on labor costs underscores the importance of engaging the academic community in addressing this issue. Leveraging technological advancements, particularly the development of electronic patient records, offers a promising avenue for effectively monitoring sensitive cost-related data. However, this necessitates interdisciplinary collaboration and the proper training of economists and health scientists. By fostering such cooperation, researchers can unlock a multifaceted approach to addressing the challenges associated with labor costs and contribute to informed decision-making in healthcare. Estimating the indirect economic effects of employees absent due to COVID-19 contributes to understanding the real cost of the pandemic for businesses and social security.

This study has a few limitations, primarily its retrospective design and focus on retail employees in the private sector in Central Greece. Another important limitation is that the researchers have not addressed potential biases in sick leave reporting and sectoral variations. Thus, while the findings provide insight into the situation among retail workers, indirect costs may vary by sector, economic conditions, and government wage policies. Future studies should control for vaccination status and inflation-adjusted wages. Upcoming investigations must also compare multiple employment sectors to minimize selection biases, examining indirect costs across various industries and including a broader range of economic variables to enhance generalizability.

## Conclusions

Our findings highlight a significant rise in labor costs for both the Hellenic Social Security Institution and organizations due to the lost productivity resulting from COVID-19, and this study represents the first attempt to quantify productivity losses and indirect costs related to this specific population in the country. Future research endeavors should delve into clinical, demographic (gender and age), and societal factors influencing COVID-19-related productivity declines to devise effective prevention and support measures for employees during public health crises. Sector-specific impacts must also be evaluated. These investigations would facilitate the implementation of informed strategic policies for resource allocation and enhance our understanding of the broader implications of the pandemic on the workforce and the economy.
